# Two New Fucose-α (1–2)-Glycans Assigned In The Healthy Human Brain Taking The Number To Seven

**DOI:** 10.1038/s41598-019-54933-1

**Published:** 2019-12-11

**Authors:** Nathan Tosh, Scott Quadrelli, Graham Galloway, Carolyn Mountford

**Affiliations:** 1 Translational Research Institute, Woolloongabba, Queensland 4024 Australia; 2 School of Clinical Sciences, Faculty of Health, Queensland University of Technology, Brisbane, QLD 4000 Australia; 3 Princess Alexandra Hospital, Department of Radiology, Woolloongabba, Queensland 4024 Australia

**Keywords:** Brain imaging, Magnetic resonance imaging

## Abstract

Fucosylated glycans are involved in the molecular mechanisms that underpin neuronal development, learning and memory. The capacity to study the fucose-α(1–2)-glycan residues noninvasively in the human brain, is integral to understanding their function and deregulation. Five fucose crosspeaks were assigned to fucosylated glycans using *in* *vivo* two-dimensional magnetic resonance Correlated SpectroscopY (2D L-COSY) of the brain. Recent improvements encompassed on the 3T Prisma (Siemens, Erlangen) with a 64-channel head and neck coil have allowed two new assignments. These are Fuc VI (F2:4.44, F1:1.37 ppm) and Fuc VII (F2: 4.29, F1:1.36 ppm). The Fuc VI crosspeak, close to the water resonance, is resolved due to decreased T1 noise. Fuc VII crosspeak, located between Fuc I and III, is available for inspection due to increased spectral resolution. Spectra recorded from 33 healthy men and women showed a maximum variation of up to 0.02 ppm in chemical shifts for all crosspeaks.

## Introduction

α-L-fucose residues, like α-L-*N*-acetyl neuraminic acid residues, are usually located at the non-reducing terminal position in an oligosaccharide chain. The intrinsic variability of the glycan structures enables sugars to encode specific information, which is recognised by receptors, and translated into a specific biological process. This fine-tuned structure-function relationship and the slight modifications on the structure of the glycan, influence their interaction with receptors and change the specific biological response.

To date, five fucose-α(1–2)-galactose sugars (glycans) and free α-L-fucose substrate have been assigned *in vivo* in the human brain using the 2D L-COSY pulse sequence^1^. These were the first new assignments in the field of neurospectroscopy in over two decades, made possible in part by improved magnet stability and by new coil technology.

The fucose-α(1–2)-galactose sugars (herein referred to as Fuc I to Fuc VII)^1^ are generally expressed as terminal saccharide glycoproteins and glycolipids^2^. The cross peaks Fuc I to Fuc VII reflect the scalar coupling between the C6-methyl groups and the protons attached to C5 of individual terminal fucose residues. They were initially identified in the literature by their structures and conformational differences found in the blood group antigens carrying terminal fucose regions^3,4^. This literature may explain the wide range of chemical shifts of the fucose-α(1–2)-glycans observed in the human brain^1^. Fucosylated glycans were shown to be important in malignancy playing a pivotal role in haematogenous metastasis^5^. These early studies on isolates of these glycans demonstrated that the MR spectral characteristics, in particular, the chemical shifts, can be altered according to the type of oligosaccharide and the point at which the fucose is incorporated^6^. Additionally, the chemical shifts of the fucose on these glycans can be changed depending on microenvironment^7^.

Fucosylated glycans are synthesised by a wide variety of fucosyltransferases. Fucosylation includes terminal fucosylation and core fucosylation. In the brain, fucose is thought to be contained within the synapsin proteins^8^, which are considered to regulate the release of neurotransmitters at the synapse^9^ with the fucosylation preventing the rapid degradation of these proteins^8^. Revest *et al*. (2010) found that blocking the fucosylation of synapsin Ia/Ib inhibits the glucocorticoid-mediated increase in stress-related memories^10^ in the hippocampus. Further to this, neuronal glycan proteins modified with fucose have been reported to be integral in learning and memory^8^ and are implicated in synaptic plasticity.

Post-traumatic stress disorder (PTSD) has been shown to affect the fucose-α(1–2)-glycans in the human brain^11^ using the 2D L-COSY protocol. Statistically significant increases were reported in the spectral region containing crosspeaks from substrate fucose, as well as in the terminal fucose on the glycans, which showed a 31% increase compared to the healthy control cohort. There was a 48% and 41% increase in fucosylated glycan Fuc IV, and Fuc VI, respectively^11^.

The purpose of this current study was two-fold. First, we aimed to determine the chemical shift variation and average peak volumes for each fucose crosspeak from a cohort of 33 healthy participants. Second, we aimed to characterise if improved shimming capability, reduced magnet drift or improved water suppression, would produce 2D L-COSY spectra with improved signal to noise ratio and spectral resolution in the region where the fucosylated glycans resonate.

## Results

### Comparison of water signal contamination in the human *in vivo* COSY recorded on the TRIO and the Prisma

**Figure 1:**
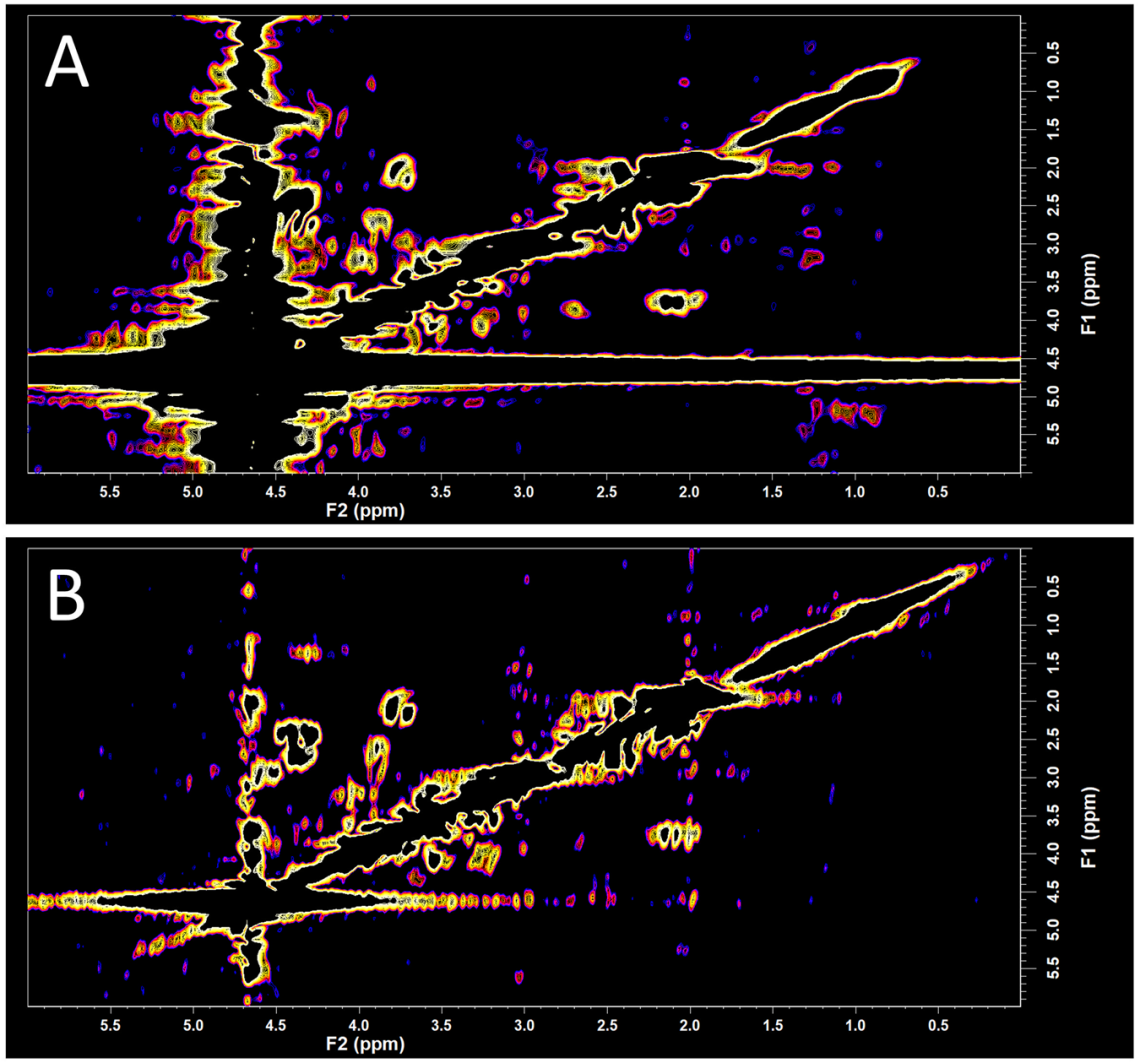
A comparison of *in vivo* 2D L-COSY spectra obtained from different 3T MR scanners utilising differenct coils. (**A**) 2D L-COSY spectra obtained on a Siemens TRIO with an eight-channel head and neck coil is shown in where the band of T1 noise around the water signal is seen to cover approximately 0.5 ppm. (**B**) The same COSY protocol collected with a Siemens Prisma with a 64 channel head and neck coil where minimal T1 noise from the water signal permits a closer inspection of the region where the fucosylated glycans resonate.

A typical 2D L-COSY spectrum obtained on a Siemens TRIO with an eight-channel head and neck coil is shown in Fig. 1A where the band of T1 noise around the water signal is seen to cover approximately 0.5 ppm. The same 2D L-COSY protocol collected with a Siemens Prisma with a 64 channel head and neck coil is shown in Fig. 1B where minimal T1 noise from the water signal band is seen to cover approximately 0.1 ppm, which permits a closer inspection of the region where the fucosylated glycans resonate.

### Reduction in acquisition time

**Figure 2:**
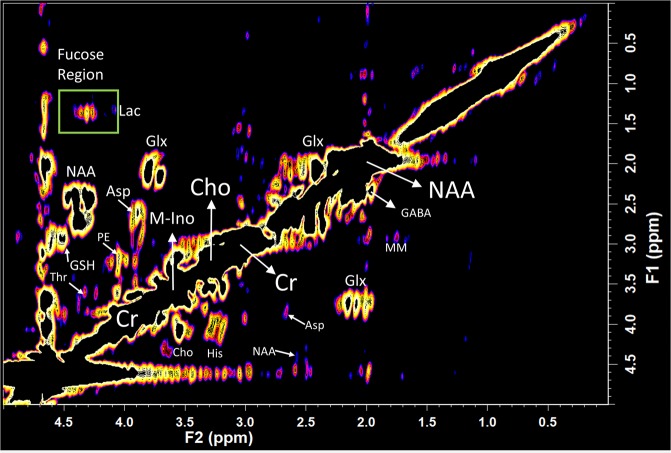
A typical *in vivo* L-COSY acquired from the PCG using a 3 T (Prisma) equipped with a 64 channel head and neck coil is shown expanded (F2: 0–5 ppm; F1: 0–5 ppm) to inspect regions of interest. The acquisition parameters were voxel size 30 × 30 × 30 mm^3^, increment size 0.8 ms, increments 96, 8 averages per increment, TR 1.5 sec, total experimental time 19:12 min, acquired vector: 1024 points, acquisition time: 512 ms, spectral width in F2: 2000 Hz, spectral width in F1: 1250 Hz. Abbreviations: N-acetyl aspartate (NAA), choline (Cho); creatine (Cr); glutamate and glutamine together (Glx); aspartate (Asp); myo-inositol (m-Ino); histidine (His); lactate (Lac); γ-aminobutyric acid (GABA); macromolecule (MM); glutathione (GSH); threonine (Thr); phosphoryl ethanolamine (PE). The region highlighted by the green box contains the crosspeaks assigned to the fucosylated glycans and is expanded in Fig. 3 as both a contour plot and 3D plot of the region where the fucosylated glycans resonate.

**Figure 3:**
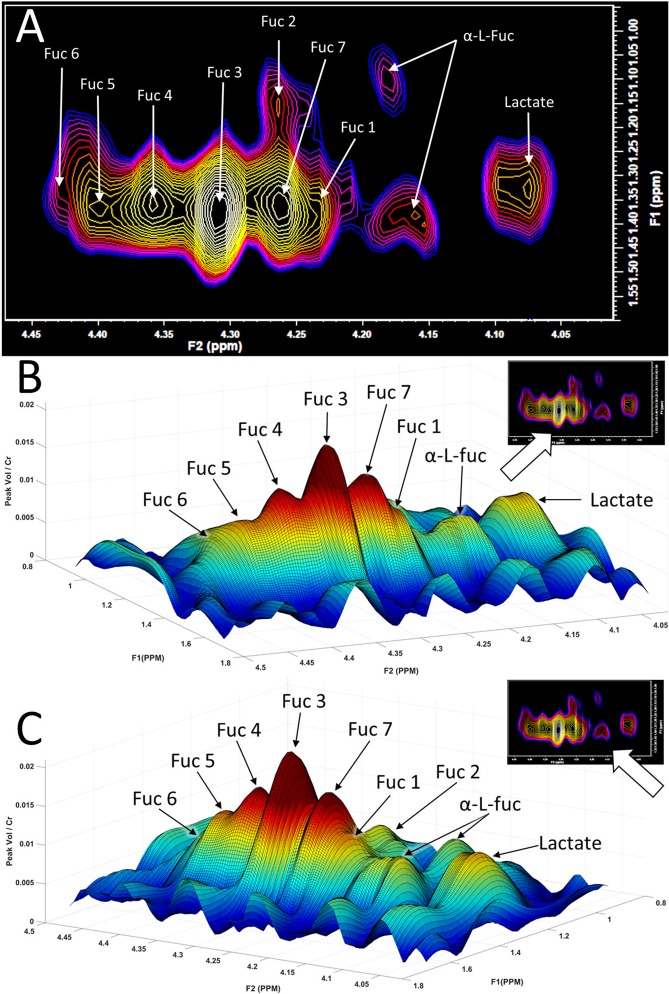
Different views of the fucose region obtained using *in* *vivo* using 2D L-COSY. (**A**) Expanded region of the L-COSY spectrum shown in Fig. 2 (F2: 4–4.5 ppm, F1: 0.95–1.6 ppm), with the assignments of Fuc I to Fuc VII denoted. α-L-fucose resonances and lactate are also visible. (**B**) 3D plot of the fucose region with the orientation as shown in the inset. The crosspeaks visible are denoted Fuc I to Fuc VII α-L-fucose I. Note Fuc I is partially visible in this projection. Fuc II and α-L-fucose I are not visible in this projection. (**C**) Fuc II and α-L-fucose I are identifiable when rotating the 3D surface plot by 90 degrees to the right.

A typical 2D L-COSY spectrum is shown in Fig. 2, acquired from the Posterior Cingulate Gyrus (PCG) in a healthy human brain at 3 T. The 1.5 second TR and 8 averages per increment reduced scan time to 19 minutes with a clear reduction in T1 noise associated with the water signal. The spectral region (F2: 4.1–4.5 ppm, F1:1.1–1.6 ppm) is expanded in Fig. 3A and shown in Fig. 3B,C as a three-dimensional plot, taken from two different viewpoints to allow for visual inspection of the region containing the fucose crosspeaks.

### Comparison of neurochemical data recorded on the prisma and TRIO scanners

The 2D COSY data recorded from 33 healthy controls were analysed and compared to those published previously using a TRIO equipped with an 8 channel head coil^1^. It can be seen in Figs. 2 and 3 that two new crosspeaks are recorded and assigned, the first at F2: 4.44 ppm-F1: 1.37 ppm and denoted Fuc VI, and the second at F2: 4.29 ppm-F1: 1.36 ppm and denoted Fuc VII. A summary of all crosspeaks recorded using the new acquisition parameters is shown in Table 1 and compared to those previously published^1^. No α-L-fucose crosspeaks were recorded in 10 of the 33 healthy subjects.

**Table 1:** Assignments and chemical shifts in the spectral region F2:4.1–4.5 ppm, and F1: 0.9–1.7 ppm from the 2D L-COSY spectra of the healthy human brain.

Crosspeak Name	TRIO Assignments[2] (F2 - F1, pm)	PRISMA Assignments (F2 - F1, ppm)	PRISMA 95% C.I. (F2 - F1, ppm)
Fuc I	4.25–1.38	4.25–1.36	±0.002 & ±0.009
Fuc II	4.28–1.14	4.28–1.13	±0.004 & ±0.01
Fuc III	4.31–1.37	4.31–1.36	±0.003 & ±0.007
Fuc IV	4.36–1.36	4.36–1.36	±0.003 & ±0.009
Fuc V	4.40–1.36	4.40–1.37	±0.003 & ±0.01
Fuc VI		4.44–1.37	±0.003 & ±0.01
Fuc VII		4.29–1.36	±0.007 & ±0.008
α-L-fucose II	4.21–1.16	4.21–1.14	±0.005 & ±0.01
α-L-fucose I	4.17–1.33	4.18–1.40	±0.005 & ±0.01

The seven published cross peak assignments[2] (TRIO and 8 channel head coil) are compared with nine crosspeaks recorded using a Prisma with a 64 channel head a neck coil. Confidence intervals of the ppm shift noted in the new assignments are listed in the right column.

Small differences in mean crosspeak chemical shifts were observed when comparing data from the TRIO^1^ and the Prisma (Table 1). In the F1 dimensions, there was a small difference at a lower ppm for Fuc I (−0.02 ppm), Fuc II (−0.01 ppm), Fuc III (−0.01 ppm) and α-L-fucose I (−0.02 ppm). Fuc V varied by 0.01 ppm and α-L-fucose II by 0.07 ppm, but at higher ppm in F1. In the F2 dimension, the only variation recorded is the α-L-fucose II crosspeak, which is 0.01 ppm different than previously reported^1^.

### Variability between the fucose spectral region for thirty-three healthy controls

**Figure 4:**
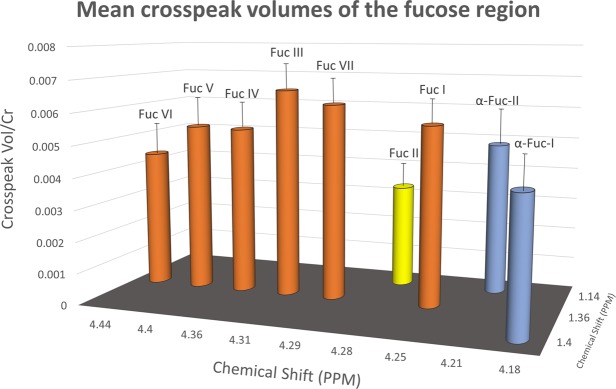
This is a schematic representation of the chemical shifts for each of the seven fucosylated glycan and substrate α-L-fucose crosspeaks. The mean crosspeak volume for all thirty-three healthy volunteers. Fuc I, IV, III, IV, V and VI (in orange) all resonate at 1.36 ppm on the F1 axis but differ in F2. The Fuc II crosspeak (Yellow) is at 1.14 ppm in F1 and is reproducibly smaller than all the other crosspeaks. The α-L-fucose substrate crosspeaks are not present in 10 out of 33 of the healthy controls but are included in Fig. 4 as the mean average from the 23 times they were present.

The average chemical shift for each assigned crosspeak and its 95% confidence interval is shown schematically in Fig. 4. The Fuc I, VII, III, IV, V and VI crosspeaks all resonate close to 1.36 ppm on the F1 axis but differ in F2 0.01–0.02 ppm. The Fuc II crosspeak is at 1.14 ppm in F1 and is reproducibly smaller than all the other crosspeaks in the healthy brain. Mean peak volumes referenced to Creatine for the seven fucosylated glycans are shown in Fig. 4. The α-L-fucose substrate(s), which are not present in 10 out of 33 of healthy controls, are also included in Fig. 4 as the mean peak volumes that were present.

## Discussion

Glycans play an essential role in several physiological and pathological processes^2,12,13^. Understanding the molecular details of glycan function, particularly *in vivo*, has been slow compared with the pace of the protein and nucleic acid research.

The initial *in vivo* assignments of these fucosylated glycans were made on a TRIO scanner equipped with an eight-channel head coil^1^. However, using this older scanner, the inspection of those fucose crosspeaks close to the water was impeded due to the large residual water signal. Also in the current study, acquired using a Prisma scanner equipped with 64 channel head and neck coil, there was a decrease in noise in the F1 dimension, particularly around the suppressed water signal, which enabled more consistent visualisation of the fucose crosspeaks.

The TR of the L-COSY sequence was also reduced from 1.8 to 1.5 seconds and the averages per increment from 12 to 8. The scan time was, therefore reduced from 35 to 19 minutes while maintaining the signal to noise ratio. These experimental conditions led to an improvement in the confidence interval of ppm shift in fucose crosspeaks by a factor of 10^1^.

As a consequence the improvements in spectral quality has allowed for two further crosspeaks to be assigned in the fucose spectral region F2: 4.44 ppm-F1: 1.37 ppm (Fuc VI) and F2: 4.29 ppm-F1: 1.36 ppm (Fuc VII) taking the total fucosylated glycans assigned number to seven. Two other crosspeaks, at F2:4.21 ppm-F1: 1.14 ppm; and F2: 4.18 ppm-F1:1.40 ppm respectively, previously assigned to the free substrate α-L-fucose, were able to be confirmed. Differences in chemical shift for the substrates has yet to be established. However, two current theories are (1) that one fucose substrate is being attached to a base pair as part of the synapse process; or (2) that the process is altered due to a different micro pH from surrounding molecules or microenvironment.

Small differences were recorded in mean crosspeak chemical shifts when comparing the data from the TRIO^1^ and the Prisma (Table 1). In the F1 dimension, there was a small difference at a lower frequency of the glycans of up to 0.02 ppm whereas in the F2 dimension the only variation recorded is the α-L-fucose II crosspeak, which is 0.01 ppm higher than previously reported^1^.

The α-L-fucose substrate crosspeaks are not present in 10 out of 33 of the healthy controls. It remains to be determined why these ten people do not have measurable α-L-fucose crosspeaks suggesting inter-patient variability or an unmeasured neuronal process of the free substrate in these participants. A current hypothesis is that the free substrate is generated to repopulate deregulated glycans.

The role of fucosylated glycans in the rat brain has been identified by studies using neuronal cell cultures and immunocytochemistry^12^. These studies reported that the level of fucosylation is highest at the synapse region of the neurons^13^. The fucosylated glycans found on neuron “cell A’s” synapse binds to proteins on the surface of neuron “cell B”, acting as a chemical bridge across the synapse to activate the cellular machinery in neuron “cell B”^12^. This was shown to trigger a positive feedback loop for the cells to synthesize more fucose and may provide an explanation for the recording of the two α-L-fucose crosspeaks at slightly different chemical shifts.

The literature on the structures and conformational differences exhibited by blood group antigens with terminal fucosylation is described by Van Halbeek *et al*.^14^, Klein^15^, and Bhattacharyya^16^. They collectively show that the chemical shift of the fucose is sensitive to the linkage type of local residues in the same molecule and that the shift effects in the MR spectrum result from different structural determinants in the sugar chain. They also suggest that the chemical shift effects found in the MR spectrum of the various determinants provide a reliable means of identification of the linkages, and that terminal fucose residues are sensitive to changes in the structure of the molecules as they form^17^. Thus, the fucosylated glycans recorded at F1:1.37 ppm are likely to be on a similar type of oligosaccharide as each other, albeit at different branches. This suggests that Fuc II at F1: 1.46 are located on a different type of oligosaccharide chain.

The observation that the 2D L-COSY method can record a signal from these glycans indicates that the fucose moiety is highly mobile on the MR timescale. This is consistent with both the fucose itself being terminal and with this population of fucose-α(1–2)-glycans being located within the synapse^12^. There may also be other populations that are not visible on the MR timescale, and it is known that the fucose-α (1–3)-glycans, for the most part, resonate under the water signal^16^. At this stage, it also cannot be discounted that one of these crosspeaks, closer to the water, is a fucose-α(1–6)-glycan^16^.

The capacity to monitor fucose-α(1–2) glycans *in vivo* in the human brain is an essential step in evaluating how these alter with brain function and how this is altered with pain and/or mental health disorders^1,11^. In a cohort of patients with PTSD, the 2D L-COSY identified statistically significant increases in the total spectral region containing both free substrate fucose and fucosylated glycans^11^. This was the first evidence of fucosylated glycans, reported in animals to be involved in learning and memory, to be affected in humans with PTSD.

A limitation of this study is the restriction to the posterior cingulate cortex region of the brain. However, other brain regions, such as the anterior cingulate cortex are under investigation. The achievement of the reduction in acquisition time from 35 to 19 minutes in this current study is significant, and there remains a technical challenge to reduce this time further.

## Conclusions

Using a 60 cm 3T Prisma clinical MRI equipped with a 64 channel head and neck coil and the application of *in vivo* 2D L-COSY MRS to the healthy human brain identifies seven fucose-α(1–2)-glycan species. Two additional crosspeaks are assigned to the substrates, α-L-fucose I and α-L-fucose II. The capacity to study the fucose-α (1–2)-glycan residues noninvasively in their native environment is an important step towards understanding the function and development of the healthy human brain and how these chemicals may alter with varying pathological conditions such as head injury, pain and the already reported findings in mental health conditions.

## Materials and Methods

### Ethical approval

Institutional ethics approval was received from Hunter New England Area Health Ethics, Australian Defence Health Research Ethics Committee, Queensland Health Metro North and Metro South Human Research Ethics Committees (HREC/16/QPAH/522). All elements of this study were carried out in accordance with approved guidelines and regulations from relevant institutional and governance bodies. Informed consent was obtained from all participants in the study.

### Participant recruitment

Participants in this prospective study were 33 healthy adults (Male = 24, Female = 9) aged between 21–57 years (Mean = 36.2) recruited from the general public via newspaper, social media advertisements or curated lists of volunteers for research from educational institutions. The inclusion criteria were: no current Axis-1 disorders detected through the Diagnostic Statistical Manual (DSM)-V, as assessed by the Structured Clinical Interview for DSM V (SCID), no lifetime history of a mood or anxiety disorder, no significant head injury, no current or past history of a central neurological disease, no current alcohol or drug dependencies, no current use of benzodiazepines, anticonvulsants or mood stabilisers, no current pregnancy or other contraindication to MRI scanning. All participants were screened by a registered clinical psychologist before the MRI session and excluded if the criteria mentioned above were not met.

### MR imaging and spectroscopy

All scans were performed on a 60 cm bore, 3T Prisma scanner (Siemens, Erlangen, Germany, software version VD13D or VE11C) with a 64-channel head and neck coil (Siemens, Erlangen) at one of three sites: Hunter Medical Research Institute (NSW, Australia), Herston Imaging Research Facility (QLD, Australia), or the Princess Alexandra Hospital (QLD, Australia).

### Structural imaging

For anatomical morphometry and voxel placement, a three-dimensional T1 weighted MPRAGE was acquired (TR/TE = 2530/1.7 ms, 12° flip angle, FOV = 256 × 256 mm, voxel size 1 mm^3^, NEX 4, IPAT = 3, acquisition time = 4 mins), and reconstructed in all planes for accurate MRS voxel placement.

### Optimising 2D L-COSY sequence

Data reported previously by Mountford *et*. *al*.^1^ were collected from a 3 T Siemens TRIO using an eight-channel head coil. In that study, a TR of 1.8 sec and 12 averages per increment was used to acquire the 2D L-COSY spectrum with a scan time of 35 minutes. Our current study aimed to decrease the T1 noise around the water signal. Hence an effort was made specifically to reduce the scan time.

2D L-COSY data were acquired from a 3 cm^3^ voxel located in the PCG. The carrier frequency was set at 2.4 ppm, TR 1.5 s; water suppression using WET; spectral width of 2000Hz; increment size of 0.8 ms in 96 T1 increments (giving an indirect spectral width of 1250 Hz); 8 averages per increment and 1024 data points. Acquisition time was 19 minutes. Shimming adjustments were undertaken on each scan by invalidating the automatic B0 field mapping technique supplied by the vendor. The automatic shim process was then performed followed by the manual adjustment of the shim gradients in the X, Y and Z directions to achieve a full width half maximum (FWHM) of the water peak between 12–15 Hz.

### 2D L-COSY data analysis

Raw data were pre-processed in Matlab^18^. The signal was combined from multiple channels, rows concatenated into a 2D matrix and reformatted. The resulting file was transformed in 2D using FelixNMR^19^, a specialised 2D NMR processing software. The post-processing parameters used in Felix were: F2 domain (skewed sine-squared window, zero-filling to 2048 points, magnitude mode), F1 domain (sine-squared window, linear prediction to 96 points, zero-filling to 512 points, magnitude mode). In Felix, each prominent diagonal and cross peak was selected and integrated to determine the peak chemical shift, intensity, and volume. These values were internally referenced using the total creatine methyl diagonal peak at 3.02 ppm. Peak and crosspeak assignments were manually adjusted, to ensure the region of integration was centred on the peak, then exported for further analysis.

The region of interest (F1 = 0.9–1.7 ppm; F2 = 4.1–4.5 ppm), referred to as the ‘fucose region’ was plotted, for all healthy volunteers, both as a contour plot (FelixNMR) and as a three-dimensional surface plot in Matlab^18^. Crosspeaks were identified in MATLAB by rotating the surface plot in all dimensions. The crosspeaks identified in the 3D surface plot were cross-referenced against the Felix contour plot to assign peak intensity co-ordinates. The average chemical shift, in ppm, of each crosspeak position from the 33 healthy volunteers was then calculated.

### Statistics

Basic descriptive statistics including means, standard deviations and confidence intervals were calculated for each of the fucose crosspeaks.

## Data Availability

The data that support the findings of this study are available on request from the corresponding author CM. The data are not publicly available due to privacy and consent concerns raised by ethical boards.
